# Humidity control in a closed system utilizing conducting polymers†

**DOI:** 10.1039/c8ra01776j

**Published:** 2018-04-03

**Authors:** Qingshuo Wei, Masakazu Mukaida, Wuxiao Ding, Takao Ishida

**Affiliations:** Nanomaterials Research Institute, Department of Materials and Chemistry, National Institute of Advanced Industrial Science and Technology (AIST) 1-1-1 Higashi Tsukuba Ibaraki 305-8565 Japan; AIST-UTokyo Advanced Operando-Measurement Technology Open Innovation Laboratory (OPERANDO-OIL), National Institute of Advanced Industrial Science and Technology 1-1-1 Higashi Tsukuba Ibaraki 305-8565 Japan qingshuo.wei@aist.go.jp mskz.mukaida@aist.go.jp; Precursory Research for Embryonic Science and Technology (PRESTO), Japan Science and Technology Agency 4-1-8 Honcho Kawaguchi Saitama 332-0012 Japan

## Abstract

In this study, we demonstrate that conducting polymers could be ideal materials for continuously managing humidity in a wide range of enclosed spaces. We demonstrate a simple battery-driven humidity control unit to manage the humidity in a closed environment and studied humidity-responsive nanocapsules using Zn-coordinated lipid nanovesicles. This study not only promises new applications for conducting polymers but also provides an easy approach for fabricating chambers with a controlled environment, which are often used by physicists, chemists, and biologists.

## Abstract 2

In this study, we demonstrate that conducting polymers could be ideal materials for continuously managing humidity in a wide range of enclosed spaces.

## Graphical abstract

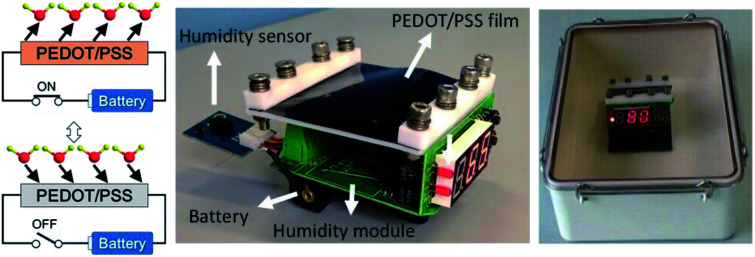

## Introduction

The control of relative humidity (RH) levels in enclosed spaces is not only important for laboratory-scale experiments in physics,^1^ chemistry,^2^ and biology^3^ but also in fields of study such as agriculture and food storage.^4^ Two methods are predominantly used to precisely control RH levels in closed system environments. One method is the gas flow method in which a dry gas that was passed through a desiccant is mixed with a gas that has been passed through water. By controlling the ratio between the two gases, the humidity can be controlled.^5^ One of the advantages of this method is that an equilibrium can be quickly established (*i.e.*, within several minutes), and the changes in the humidity can be continuous. However, this system is relatively complicated and bulky. Aside from the gas supply, a number of different moving parts and valves are required for the construction of the equipment required for this method. Furthermore, the method does not create a closed system, which limits its applicability to laboratory-scale experiments such as gas analysis. The second method predominantly used to precisely control humidity involves the use of saturated salt solutions such as lithium chloride or magnesium chloride.^6^ This method is highly accurate and relatively simple to perform, because the concentration of a saturated solution is fixed and the vapor pressure can be precisely determined for different temperatures. Because of its high accuracy, this method is used for the certification of humidity sensors. However, this method can lead to salt contaminating a closed system, which is detrimental for laboratory-scale experiments. Furthermore, the humidity value that can be obtained using this method are discontinuous, as the salt solution has to be changed so that different humidity values can be obtained. This can result in it taking several hours for an equilibrium to be achieved.

In this paper, we report on a simple approach that can be used to precisely control RH levels (from 20% to 90%) in a closed system in a continuous manner; this is achieved through the use of conducting polymer films. This approach involves no moving parts or chemicals that could contaminate an experimental environment, and an equilibrium is achieved within several minutes. By combining a humidity switch module using a printed circuit board, we were able to demonstrate a compact battery-driven humidity control unit that was less than 7 cm × 7 cm in size. We found that this could be used to control the humidity in an environmental chamber with a capacity of over 1 L. We also demonstrated the morphology change of humidity-responsive nanocapsules using Zn-coordinated lipid nanovesicles, which could potentially contribute to the controlled release of chemicals from nanocapsules under different humidity conditions.

## Results and discussion

**Fig. 1:**
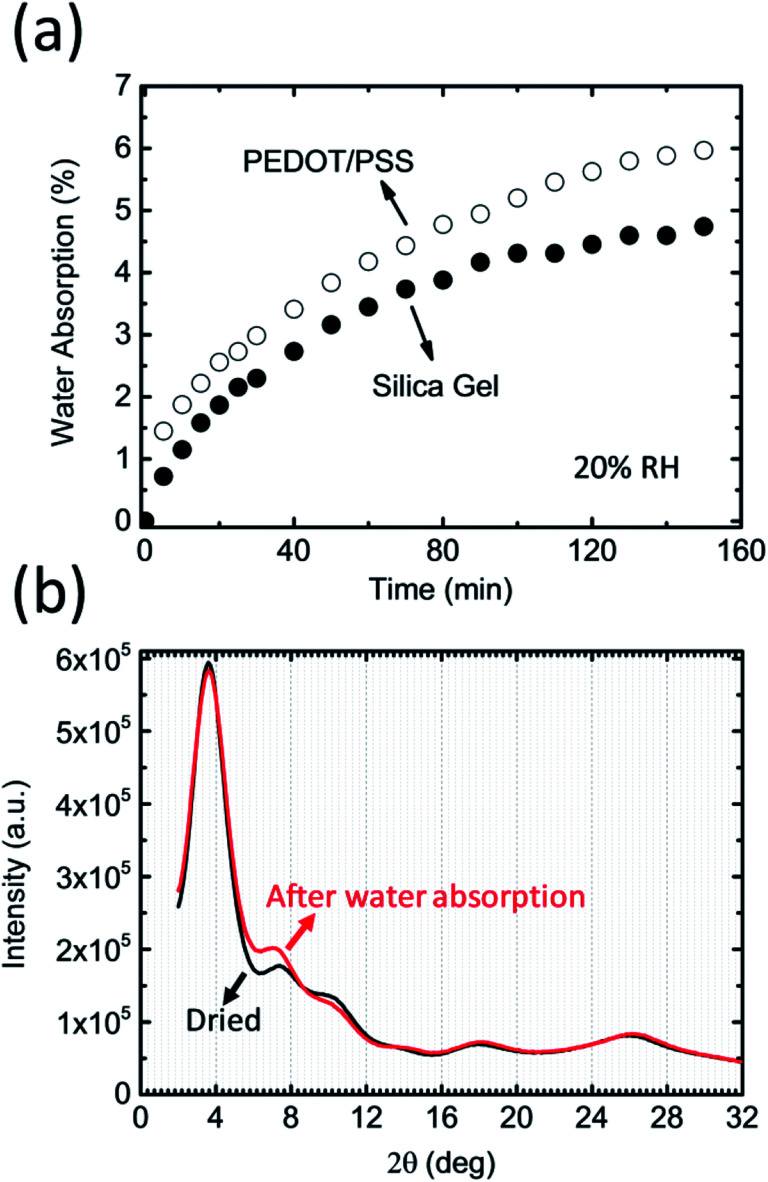
(a) Percentage of the water absorbed by a PEDOT/PSS film (open circles) and silica gel (closed circles) as a function of time at a humidity of 20%. (b) XRD patterns of the PEDOT/PSS film before (black) and after (red) water absorption from the air.

Our proposal utilizes the conducting polymer poly(3,4-ethylenedioxythiophene)/polystyrene sulfonate (PEDOT/PSS) to control the humidity in a closed system. PEDOT/PSS has attracted a lot of attention owing to its potential for use in applications such as energy storage, energy conversion, and flexible electronics.^7^ It is well known that PEDOT/PSS films readily absorb water from the air.^8,9^ The water uptake of PEDOT/PSS films is reported to have a negative effect on the stability and reproducibility of organic electronic devices because many organic semiconductors and metal electrodes are sensitive to moisture.^10^ However, the extremely strong hygroscopic behavior of PEDOT/PSS films has not attracted much attention. If a freshly prepared free-standing PEDOT/PSS film is placed into a glovebox that has 20% humidity, the weight of the film will increase over time and eventually reach saturation. It has been suggested that this film can absorb water even in very low-humidity conditions and that the hygroscopicity of PEDOT/PSS films could be stronger than that of desiccant silica gels (Fig. 1a). As such, the measured moisture absorption rate could be greater than 8% at an RH of 20%. A simple calculation indicates that 1 g of PEDOT/PSS film (with a volume of *ca.* 0.7 cm^3^) can absorb roughly 80 mg of water at 20% RH. This value is equivalent to the amount of water contained in 3.4 L of air at 100% RH (the saturated water vapor pressure at 25 °C is roughly 3.2 × 10^3^ Pa). Using the ideal gas law, we find that the volume 
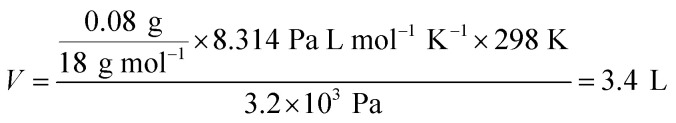
. In other words, the humidity of a closed system can be controlled using a PEDOT/PSS to gas volume ratio of only 1/5000. This calculation suggests that if one can control the amount of water present in a PEDOT/PSS film, an ideal material for managing the humidity in a closed system could be created.

The largest difference between PEDOT/PSS films and other desiccants is their high electrical conductivity.^11–16^ As a benchmark conducting polymer, PEDOT/PSS films exhibit among the highest electrical conductivities (*i.e.*, close to 1000 S cm^−1^) and the electrical conductivity value is less sensitive to the humidity (the amount of water in the film).^8^ This is because the PEDOT nanocrystals form a layered structure. The spacing between the layers could change before and after the water absorption, but the crystal ordering and crystal size don't significantly affect by the amount of the water in the film (Fig. 1b). If a voltage is applied to the PEDOT/PSS film, an electric current passes through the PEDOT nanocrystals, and the water in the film may desorb owing to joule heating. The idea underpinning this work is that the amount of water in a PEDOT/PSS film can be controlled by regulating the current density passing through the film.

**Fig. 2:**
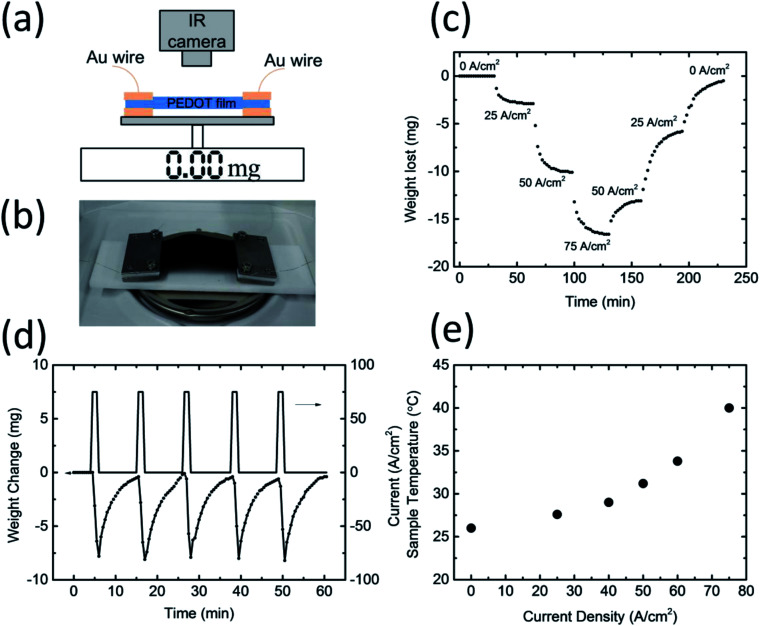
(a) Schematic and (b) image of the procedure for measuring the change in weight of the PEODT/PSS film during a current flow. (c) Loss in weight of the PEDOT/PSS film as a function of time for different current flows. (d) Change in weight of the PEDOT/PSS film for a pulse current density of 75 A cm^−2^. The on-time lasted 1 min, and the off-time lasted 10 min. (e) Sample temperature plotted as a function of the current density.

In order to confirm whether water absorption and desorption occur in PEDOT/PSS films during current flow, we prepared a free-standing film with a thickness of *ca.* 75 μm. The film was cut into a 2.6 cm × 5.0 cm rectangle, and a gold wire with a diameter of 150 μm was mounted onto the sample. The weight of the film was around 140 mg, and the electrical conductivity of the film was *ca.* 800 S cm^−1^. The sample was put onto an analytical balance, and an infrared camera was used to measure the temperature of the sample (Fig. 2a and b). The experiment was conducted at room temperature at an RH of 55%. As shown in Fig. 2c, when a constant current density of 25 A cm^−2^ (0.5 A) was applied to the film, its weight started to decrease and became saturated at *ca.* 3 mg. This can be attributed to the desorption of water from the polymer film. A further *ca.* 10 mg was lost when the current density was increased to 50 A cm^−2^ (1.0 A), and 18 mg in total was lost for a current density of 75 A cm^−2^ (1.5 A). Interestingly, the weight lost as a constant current density does not keep decreasing but will saturate at a constant value. Once a larger current was applied to the sample, the weight began to further decrease. However, when the current density decreased, the weight began to increase; this suggests that the PEDOT/PSS film began to absorb water from the environment. The weight of the film did not increase continuously, but rather became saturated at a certain value depending on the current density. At a current flow of zero, the weight of the film reverted to the initial value shown in Fig. 2c. This result suggests that the amount of water inside the film can be readily controlled through the tuning of the current density. Note that the saturated weight of the sample differed slightly at the same current level when the current density was increased and when it was decreased. This thermal hysteresis may be related to the change in the resistivity of the PEDOT/PSS film during the heating and subsequent cooling process. When the current density increased, the resistivity of the PEDOT/PSS film increased as the water was baked out of the system.^17^ Note that the carrier mobility in the PEDOT component of the film was partly limited by the columbic interaction of the PSS^−^. This interaction may have screened the water molecules owing to the high dielectric constant. Once the water was baked out, the carrier mobility decreased, and the resistivity increased. For the same current density passing through the film, the sample temperature was higher during the cooling process owing to a larger resistivity. As a result, the amount of the water uptaken from the air is smaller. At a current density of zero, the temperature of the film should be the same as that of the room temperature, which would result in the weight of the film reverting to its initial value.

In order to study the stability and reproducibility of water desorption and absorption of the PEDOT/PSS films during current flow, we applied a 75 A cm^−2^ pulse current at a duration of 60 s to the films. The weight of the films quickly decreased before returning to their initial weight after *ca.* 10 min. The absolute amount of water desorbed during the pulse current and the recovery time remained almost constant, which suggests that the PEDOT/PSS films are very stable. The water desorption of the PEDOT/PSS films during the current flow was faster than the water absorption from the atmosphere. Fig. 2e shows the change in the temperature of the samples during the current flow. As would be expected, the temperature increased as the current increased, with the temperature increasing by approximately 1 °C for a current density of 25 A cm^−2^, 5 °C for a current density of 50 A cm^−2^, and 14 °C for a current density of 75 A cm^−2^. Although a higher current density can desorb a greater amount of water, it is not preferable for humidity control in a closed system because it could raise the temperature of the system. In order to avoid any unwanted temperature changes from occurring during humidity control, we set the current density to being smaller than 40 A cm^−2^ in our experiment.

**Fig. 3:**
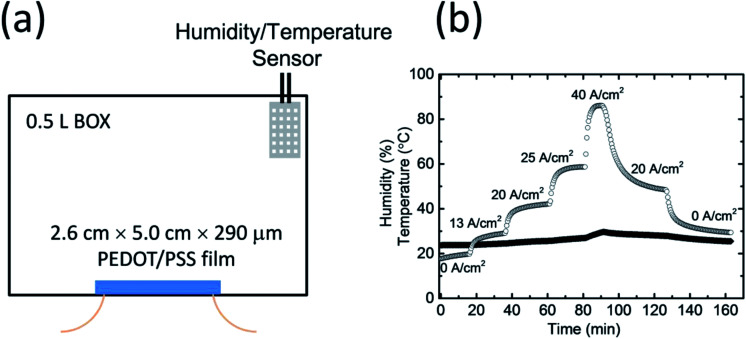
(a) Schematic of the humidity control device that utilizes a PEODT/PSS film. (b) The humidity (open circle) and temperature (closed square) in the closed system as a function of time for different current flows.

A PEDOT/PSS film whose dimensions were 2.6 cm × 5.0 cm × 290 μm was fixed inside a sealed box that had a volume of 0.5 L (Fig. 3a). The humidity and temperature in the box were recorded by a highly accurate temperature and humidity data logger. The box was initially filled with dry air, and a humidity value of *ca.* 20% was maintained as a starting point. As shown in Fig. 3b, when an electric current flowed through the PEDOT/PSS film, the humidity in the box increased; this was because of water desorbing from the film. After reaching an equilibrium, the saturated humidity in the box depended on the current density. When a constant current density of 13 A cm^−2^ was applied to the film, the humidity increased and became saturated at *ca.* 30%. The saturated humidity was *ca.* 42% at a current density of 20 A cm^−2^, 62% at a current density of 25 A cm^−2^, and over 86% at a current density of 40 A cm^−2^. When the current density decreased from 40 to 20 A cm^−2^, the saturated humidity dropped to 49% owing to the water absorption of the PEDOT/PSS film. When we stopped applying the voltage to the PEDOT/PSS film, the humidity in the box dropped below 30% after 30 min. The change in temperature in the sealed box over the entirety of the measurement process was less than 5 °C. This result is consistent with the observation of the change in weight of the film shown in Fig. 2c.

**Fig. 4:**
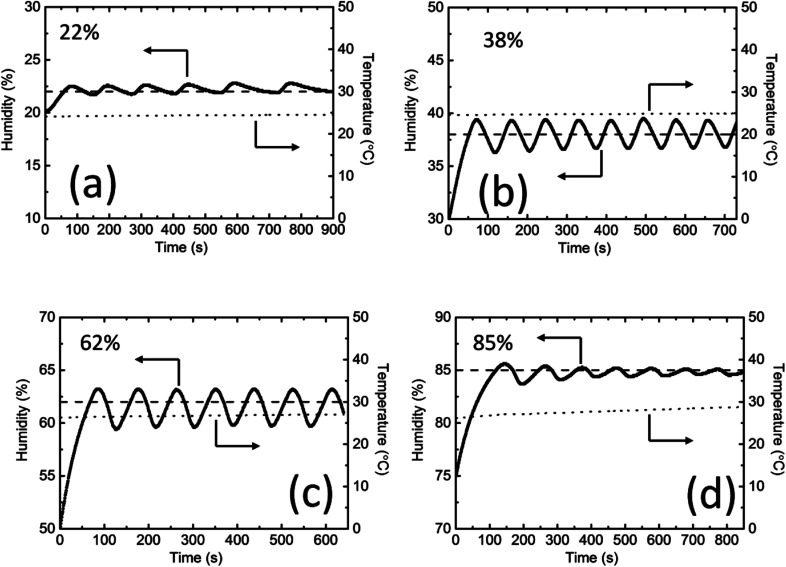
On/off humidity control action for different humidity values: (a) 22%, (b) 38%, (c) 62%, and (d) 85%. The heating power is either on when the humidity is below the setpoint or fully off when it is above. As a result, the humidity oscillates about the setpoint. The solid line represents the humidity that was measured, and the dotted line represents the temperature that was measured. The setpoint is shown as a dashed line.

Fig. 3b shows that humidity control in a closed system can be achieved by controlling the current density applied to the PEDOT/PSS films. In practical situations, it is normally difficult to precisely set the humidity by only tuning current density. However, the fast response of the water absorption and desorption mechanisms in PEDOT/PSS films facilitates the control of the humidity in the system *via* an on/off switch; the switch will turn on the power supply to the PEDOT/PSS film when the humidity falls below a previously defined point (which we will call the “on”-point), and it will turn it off when the humidity rises above a different previously defined point (which we will call the “off”-point). We continuously conducted the humidity control experiment using a single set-up (*i.e.*, the same PEDOT/PSS film and the same sealed box); no gas exchange took place during the process. Fig. 4 shows the on/off humidity control action for different humidity settings. The rise in the humidity value that exceeded the “off”-point caused an overshoot before the humidity decreased again when the power was off owing to the water absorption by the film. The actual humidity value in the box oscillated near the set point. At a low RH of 22%, the humidity increased much faster than it decreased (Fig. 4a); that was because the film absorbed water relatively slowly in low-humidity conditions, and the off time was longer. The circle time was around 140 s. For RH values of 38% and 62%, the circle time was around 90 s (Fig. 4b and c). The time-dependent humidity change that was measured exhibited a sine wave shape. At an RH of 85%, the on-time was longer because the water absorption by the PEDOT/PSS film was faster under high-humidity conditions. The circle time was around 100 s. The cycle time over the entire range of humidity values tested was comparable with that of the gas flow method and was much shorter than when the humidity was controlled using saturated salts. The bandwidth was smaller than 4% (±2%) over the entire range of humidity values tested, which was comparable to that of the gas flow method (the accuracy of both methods relies on the sensitivity of the humidity sensor). In all of the experiments, the change in temperature for lower humidity values was less than 1 °C. At an RH of 85%, the change in temperature in the chamber was approximately 2 °C. It is known that humidity is dependent on both temperature and pressure. Our current device used resistive humidity sensors. The working temperature range of the sensors is from −40 to 85 °C under standard atmospheric pressure. If we use capacitive humidity sensors, our devices may work up to a higher temperature under a higher pressure.

**Fig. 5:**
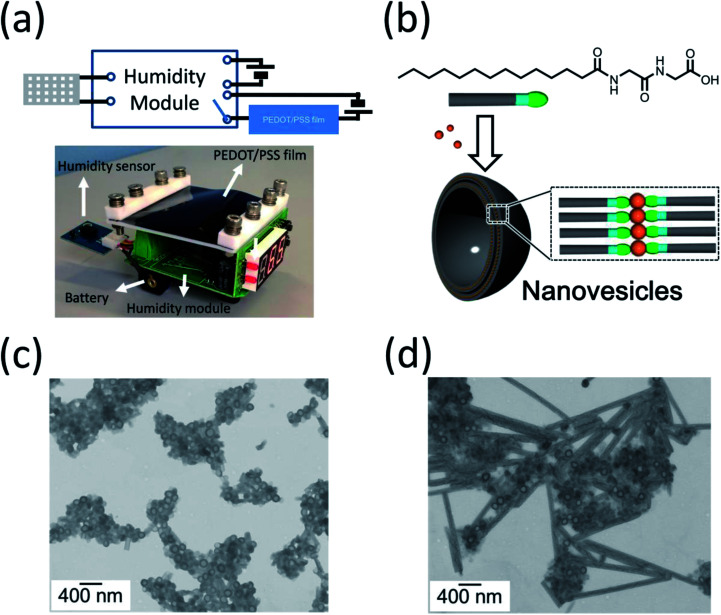
(a) Schematic diagram and image of the humidity control unit using a humidity switch on a printed circuit board. (b) Schematic illustration of the nanovesicles by mixing the glycylglycine-containing lipid with zinc acetate. STEM images of (c) as-prepared Zn-coordinated nanovesicles with 5% Tween 20 (w/w) and (d) after these vesicles have been stored at a RH of 90% for 1 day.

The compact and simple structure of our device is an advantage. The high conductivity of the PEDOT/PSS film makes the device have low power consumption, such that it could be driven using a battery. The two-probe resistivity of the PEDOT/PSS–Au wire in our set-up was less than 0.3 Ω. A 1.5 V battery can produce a current density as high as 35 A cm^−2^. We were able to use a battery-driven on/off switch on a printed circuit board to fabricate the proof-of-concept humidity controller, as shown in Fig. 5a. The size of the humidity control unit can be made to be less than 7 cm × 7 cm × 2.5 cm, which would make it compact enough to be put into an environmental chamber in a lab-scale experiment. This device could continuously operate for more than one week. A video of the humidity control device at an RH of 90% is shown in the ESI.†

The humidity control approach proposed in this study could be used in a closed system, which has the advantage that it allows us to study a controlled release of chemicals from nanocapsules, because no chemicals or gases are exchanged when the humidity is varied. The control of the release of chemicals from nanocapsules, which are usually accompanied by a morphological change in the nanocapsules under various stimuli, is important because of their applications to medicine, food, and daily care products. However, research into humidity-responsive nanocapsules is far less reported upon than conventional pH-, light-, and temperature-responsive nanocapsules.^18^ In this paper, we investigate humidity-responsive nanocapsules in a closed plastic chamber using Zn-coordinated lipid nanovesicles (Fig. 5b).^19,20^Fig. 5d shows the transmission electron microscopy image of as-prepared nanovesicles mixed with 5 wt% polyoxyethylene sorbitan monolaurate (Tween 20). The nanovesicles have a spherical structure with a homogeneous diameter of around 120 nm as well as a fine membrane structure (Fig. 5c). This structure is very stable in open air at humidity levels below 60%. After the RH was increased to 90% for one day in the closed environment, the morphology of the nanovesicles began to change; the membranes of the nanovesicles opened, their spherical structure disappeared, and the nanocapsules began to combine with one another to form nanotubes (Fig. 5d). This result suggests that chemicals encapsulated in the nanovesicles will be released in high-humidity conditions. Because our approach is able to precisely and continuously control the humidity in a closed system, it could be an ideal method for studying and optimizing the humidity-responsiveness of nanovesicles combing with gas analysis such as chromatography-mass spectrometry and *in situ* Fourier transform infrared spectrometer (FT-IR). The encapsulation of functional molecules for daily care products and agricultural chemicals and its release is a currently much researched topic.

## Conclusion

In conclusion, we have demonstrated a novel approach to controlling the humidity in a closed system with an RH between 20% and 90%. In comparison to the gas flow method and the approach that uses saturated salt solutions, there are no moving parts in our method nor are there any chemicals that will contaminate the experimental environment; furthermore, equilibrium is very quick to achieve. Owing to the high electrical conductivity of conducting polymers, our humidity control unit consumes very little power. We believe that this will allow for humidity control units that can be powered by batteries to be developed. Our proposed device could also contribute to the study of the controlled release of substances for potential future applications in daily care products or agricultural chemicals. This study not only presents new potential applications for conducting polymers but also provides a new way to make more compact, simple, and low-cost environmental chambers.

## Conflicts of interest

There are no conflicts to declare.

## Supplementary Material

RA-008-C8RA01776J-s001

RA-008-C8RA01776J-s002
