# Supplementary material for: Humidity control in a closed system utilizing conducting polymers
Source: RSC Adv. 2018 Apr 3;8(23):12540–6. doi: 10.1039/c8ra01776j (PMC9079635; doi:10.1039/c8ra01776j)
Supplement: RA-008-C8RA01776J-s002 [file RA-008-C8RA01776J-s002.pdf]

Electronic Supplementary Material (ESI) for RSC Advances.
This journal is © The Royal Society of Chemistry 2018

Supporting Information

Humidity Control in a Closed System utilizing Conducting Polymers

Qingshuo Wei[*,†,‡,§], Masakazu Mukaida*[*,†,‡], Wuxiao Ding*[†], and Takao Ishida*[†,‡]

[†]Nanomaterials Research Institute, Department of Materials and Chemistry, National Institute of Advanced Industrial Science and Technology, 1-1-1 Higashi, Tsukuba, Ibaraki 305-8565 Japan

[‡]AIST-UTokyo Advanced Operando-Measurement Technology Open Innovation Laboratory (OPERANDO-OIL), National Institute of Advanced Industrial Science and Technology, 1-1-1 Higashi, Tsukuba, Ibaraki 305-8565 Japan

[§]Precursory Research for Embryonic Science and Technology (PRESTO), Japan Science and Technology Agency, 4-1-8 Honcho, Kawaguchi, Saitama 332-0012, Japan

Chemicals. A PEDOT/PSS (Clevios PH1000) solution was purchased from H. C. Starck, and ethylene glycol (>99.5%) was purchased from TCI Chemicals. Silica gel (AMIX6UP) was purchased from ToyotaKako.

Film Preparation. The free-standing PEDOT/PSS films were prepared following the procedure outlined in our previous report.[1,2] In brief, a PEDOT/PSS solution (10 mL) containing 3 wt% ethylene glycol was added to a polystyrene case and heated on a hot plate at 40 °C. After all of the solvent had evaporated, the PEDOT/PSS film could be readily detached from the case; the thickness of the film was ca. 70 μm. The as-prepared film was then cut into

a 2.5 cm × 5.0 cm rectangle using a precision film cutter. A humidity detection switch (MH13001) was used to fabricate the compact humidity control unit.

Characterization. The weight of the film was measured using a semi-micro analytical balance (MS105DU, Mettler Toledo). The thickness of the film was measured using a high-resolution digimatic measuring unit (VL-50-B, Mitsutoyo). The conductivity was measured by a four-probe conductivity test meter (MCP-T600, Mitsubishi Chemical Corp.). The current was controlled using a source measure unit (Yokogawa GS820). The humidity and temperature were monitored using a memory data logger (LR8400, Hioki) with a Z2000 humidity sensor and a K-type thermocouple. The humidity sensors show linear range from 10 ～90 %RH. The error is within 5%. The surface temperature of the polymer film was monitored using an infrared camera (PI 400, Optris). All of the experiments at a humidity of 20% were conducted in a stainless vacuum glovebox (UN-800, Unico). X-ray diffraction (XRD) patterns were recorded on a Rigaku SmartLab diffractometer.

Nanovesicle Preparation. The Zn-coordinated nanovesicles were prepared by the reaction of zinc acetate (60 mg, 0.32 mmol) with a lipid (200 mg, 0.58 mmol) solution in 2 mL of ethanol for 2 h at room temperature. Subsequently, 10 mg of Polyoxyethylene Sorbitan Monolaurate (Tween 20) was added into the nanovesicle dispersion. 10 μL of the dispersion was then sampled, diluted, and dried on carbon grids so that the dispersion could be observed by scanning transmission electron microscopy (STEM, Hitachi S-4800). One of the carbon grids was stored in the aforementioned humidity chamber (90% RH), and the morphology was confirmed after an exposure duration of 1 day.

(1) Wei, Q.; Mukaida, M.; Kirihara, K.; Ishida, T.: Experimental Studies on the Anisotropic Thermoelectric Properties of Conducting Polymer Films. *ACS Macro Letters* **2014**, *3*, 948-952.

(2) Wei, Q.; Mukaida, M.; Kirihara, K.; Naitoh, Y.; Ishida, T.: Thermoelectric power enhancement of PEDOT:PSS in high-humidity conditions. *Applied Physics Express* **2014**, *7*, 031601.
